# Early Protein Intake Influences Neonatal Brain Measurements in Preterms: An Observational Study

**DOI:** 10.3389/fneur.2020.00885

**Published:** 2020-08-26

**Authors:** Gianluca Terrin, Maria Chiara De Nardo, Giovanni Boscarino, Maria Di Chiara, Raffaella Cellitti, Simona Ciccarelli, Corinna Gasparini, Pasquale Parisi, Matteo Urna, Benedetta Ronchi, Alessia Russo, Giulia Sabatini, Mario De Curtis

**Affiliations:** ^1^Department of Maternal and Child Health, University of Rome La Sapienza, Rome, Italy; ^2^Child Neurology, NESMOS Department, Faculty of Medicine & Psychology, Sapienza University, Rome, Italy

**Keywords:** newborn, VLBW, enteral nutrition, parenteral nutrition, nutrition, amino-acids solution, cranial ultrasound, cerebral growth

## Abstract

**Introduction:** To limit extrauterine growth restriction, recent guidelines on nutrition of preterm neonates recommended high protein intake since the first day of life (DOL). The impact of this nutritional strategy on the brain is still controversial. We aimed to evaluate the effects of protein intake on early cerebral growth in very low birth weight newborns.

**Materials and Methods:** We performed serial cranial ultrasound (cUS) scans at 3–7 DOL and at 28 DOL in very low birth weight newborns consecutively observed in the neonatal intensive care unit. We analyzed the relation between protein intake and cerebral measurements at 28 DOL performed by cUS.

**Results:** We enrolled 100 newborns (gestational age 29 ± 2 weeks, birth weight 1,274 ± 363 g). A significant (*p* < 0.05) positive correlation between enteral protein intake and biparietal diameter (*r* = 0.490^**^), occipital–frontal diameter (*r* = 0.608^**^), corpus callosum (length *r* = 0.293^*^, genu *r* = 0.301^*^), caudate head (right *r* = 0.528^**^, left *r* = 0.364^**^), and cerebellum (transverse diameter *r* = 0.440^**^, vermis height *r* = 0.356^**^, vermis width *r* = 0.377^**^) was observed at 28 DOL. Conversely, we found a significant negative correlation of protein intake given by parenteral nutrition (PN) with biparietal diameter (*r* = −0.524^**^), occipital–frontal diameter (*r* = −0.568^**^), body of corpus callosum (*r* = −0.276^*^), caudate head (right *r* = −0.613^**^, left *r* = −0.444^**^), and cerebellum (transverse diameter *r* = −0.403^**^, vermis height *r* = −0.274^*^, vermis width *r* = −0.462^**^) at 28 DOL. Multivariate regression analysis showed that measurements of occipital–frontal diameter, caudate head, and cerebellar vermis at 28 DOL depend positively on protein enteral intake (*r* = 0.402^*^, *r* = 0.305^*^, and *r* = 0.271^*^) and negatively by protein parenteral intake (*r* = −0.278^*^, *r* = −0.488^*^, and *r* = −0.342^*^).

**Conclusion:** Brain development in neonatal life depends on early protein intake. High protein intake affects cerebral structures' measurements of preterm newborn when administered by PN. Positive impact on brain development encourages the administration of recommended protein intake mainly by enteral nutrition.

## Introduction

Extrauterine growth restriction (EUGR) frequently occurs in preterm neonates during the first weeks after birth (1). The growth failure in early life is, in turn, associated with an increased risk of neurological impairment (1). A number of studies demonstrated that high protein intake may limit EUGR (2). Thus, current guidelines for preterm newborns recommend the administration of high macronutrient doses since the first hours of life, through parenteral route (3, 4). The effect of this nutritional strategy on brain growth is widely debated. A limited number of studies investigated the impact of different nutritional practices on brain volume, using magnetic resonance imaging (MRI) at term equivalent age (TEA) (5). Considering inconclusive results of these studies, further research has been advocated (6). However, MRI is not easy to perform during the first weeks of life when clinical conditions of newborns are critical, even if this technique is considered the gold standard for the study of cerebral structures' measurements (7). Recently, it has been demonstrated that cranial ultrasound (cUS) can be reliably used to monitor cerebral growth in preterm infants (6, 8–11). The cUS is a low-cost bedside technique, repeatable as often as necessary, available, and widely used in the neonatal intensive care unit (NICU) (8). Starting from these considerations, we aimed to study the effects of high protein supply on early cerebral volume, through serial cUS examinations in preterm neonates.

## Materials and Methods

### Study Design and Population

We designed a prospective observational study to assess the effects of protein intake on brain measures by using two-dimensional cUS in preterm neonates. All newborns with gestational age (GA) <32 weeks or body birth weight (BW) <1,500 g, consecutively admitted to the NICU of Policlinico Umberto I, La Sapienza University of Rome, were prospectively included between May 2017 and August 2019. We excluded infants with major congenital malformations, inborn errors of metabolism, congenital infections, intraventricular hemorrhage (IVH) stage ≥3, death, or transfer to other hospital before 72 h of life (12–15).

### Collection Data

Prenatal, perinatal, and postnatal data were prospectively collected for each patient in specific data form. In particular, GA, BW, gender, type of delivery, twin pregnancy, antenatal steroid administration, Apgar score at the 1st and 5th min after birth, pH on cord blood at birth, body temperature at the 1st h of life, Clinical Risk Index for Babies II score (CRIB II), death, and need of invasive mechanical ventilation were recorded (16). Diagnosis of the major morbidities associated with prematurity, such as necrotizing enterocolitis (Bell stage ≥2), bronchopulmonary dysplasia, IVH, PVL, retinopathy of prematurity, and sepsis proven by positive cultures, were performed according to the standard criteria and recorded in the reporting form, as previously described (17, 18). Data on daily enteral and parenteral nutritional intake were collected during the first week of life. We collected data on parenteral nutrition (PN) complications. We measured head circumference of the newborns with a tape measure. We wrapped the tape, holding it above the eyebrows and the ears and the occipital prominence at the back of the skull, around the widest possible circumference of the head.

### Nutritional Protocol

Enteral nutrition (EN) was commenced as soon as possible after birth in a stable newborn. Minimal enteral feeding was started within 24–48 h after birth at 10–20 mL/kg per day. The amount was increased by 20–30 mL/kg every day if EN was tolerated. Maternal milk (MM) without fortifications if available has been given fresh. Whether MM was not available or sufficient, a formula specifically for preterm newborns and routinely given in our NICU was used. Donor breast milk during the study period was not available. When signs or symptoms of feeding intolerance such as emesis, vomiting, severe abdominal distension associated with ileus with visible intestinal loops, blood in the stools, or systemic disorders (i.e., apnea, bradycardia, inadequate perfusion, and hemodynamic instabilities) were observed, the EN was withheld for at least 24 h (19, 20). Preterm MM was assumed to contain 65 kcal/100 mL, 1.5 g of protein/100 mL, 3.5 g of fat/100 mL, and 6.9 g of carbohydrate/100 mL. Macronutrient contents of formula and parenteral solutions were calculated based on the published manufacturers' recordings (Pre-nidina Nestlè®: proteins 2.9 g/dL, lipids 4.0 g/dL, energy 8.1 g/dL, sodium 51 mg/dL, potassium 119 mg/dL, calcium 116 mg/dL, phosphorus 77 mg/dL, iron 1.8 mg/dL, zinc 1.2 mg/dL). Parenteral nutrition was administered at birth in order to maintain adequate fluid, electrolyte, and nutrient intakes until exclusive enteral feeding (120 kcal/kg per day) was achieved. The overall fluid intake administered with enteral and PN started with 70–90 mL/kg per day and slowly increased by 10–20 mL/kg per day until reaching 150–180 mL kg per day. In PN, we administered 2 g of amino acids (TrophAmine® 6% Braun Medical Inc., Irvine, CA, USA) in the first day of life (DOL), and then we increased protein intake of 1 g/kg per day up to 4 g/kg per day, with 25 kcal per 1 g of proteins (Table S1). Glucose intake (dextrose injection 10%; Fresenius Kabi, USA) was started at 6 to 7 g/kg per day and increased of 0.5–1 g/kg per day up to 14 g/kg per day. Lipid (Smoflipd® Fresenius Kabi, USA) intake was started at 1 g/kg per day and increased of 0.5–1 g/kg up to 3.5 g/kg per day. Total energy intake was calculated based on the cumulative amount of PN and EN in kcal/kg over the early 7 days. Target dose refers to enteral plus PN; thus, we adjusted intake from PN according to the amount of EN tolerated.

### Cranial Ultrasonography Examination

The transducer frequency of sector probe (Philips EPIQ, Amsterdam, the Netherlands) was set at 5–10 MHz. Images were recorded in coronal and sagittal planes, according to standard procedure. We considered the anterior fontanel the optimal acoustic window for visualization of the supratentorial structures, whereas we used mastoid fontanel to evaluate the cerebellum and cerebellar vermis.

The cUS scans were obtained in enrolled newborns, at 3 to 7 DOL (T0) and at 28 DOL (T1), by two examiners with high training in cUS, unaware of the nutrition protocol and study aims. Each measurement was confirmed after an agreement between the two sonographers. They performed each measurement three times and then reported the mean value in a specific data form.

Cerebral structures were measured as previously described (6, 21–24). Scanning was performed at the bedside with the infant's head in supine position. With the anterior fontanel used as an acoustic window, standard views were obtained in the coronal and sagittal planes. In brief, intracranial biparietal diameter was measured in a coronal plane at the level of the foramen of Monro, and maximum intracranial occipital–frontal diameter was measured in the midsagittal plane. Maximum length of corpus callosum was measured in the midsagittal plane tracing a horizontal line between the extreme margins of the genu and the splenium. Maximum width of corpus callosum was measured in the midsagittal plane, separately for genu, body, and splenium. We visualized caudate nucleus below the floor of the frontal horn of the lateral ventricle, as a hypoechoic area located anteriorly to the caudothalamic groove. Width of the caudate head was measured in the parasagittal plane as the maximum extension of this area. Both height and width of the cerebellar vermis and transverse cerebellar diameter were measured in axial plane.

### Statistical Analysis

Data analysis was performed using IBM the Statistical Package for the Social Sciences Statistics version 22.0 (SPSS Inc., Chicago, IL, USA). We checked for normality using Shapiro–Wilk test. The mean and standard deviation or median and interquartile range summarized continuous variables. We compared categorical variable using χ^2^ test and paired and unpaired variables by *t* test or Mann-Whitney *U* test. Nutritional intake was related to the bidimensional measurements of the different brain structures and cerebral diameters collected during the first week of life (T0) and at 28 DOL (T1). We performed correlation between variables by Wilcoxon rank sum tests and by Pearson correlation. To evaluate the effects of protein intake given by PN in homogenous population, we selected newborns that were nourished mainly by PN in the first week of life. In particular, we analyzed separately patients receiving more than 70% of nutritional support by PN from 0 to 7 DOL. In this subpopulation, we calculated the percentile of total protein intake at the first week of life. Thus, we considered at high protein regimen intake patients receiving any value equal to or >50th percentile and at low protein regimen intake those receiving <50th centile of protein intake. To evaluate the effect of different protein intake on the size of cerebral structures, we compared newborns at high protein regimen intake with those at low protein regimen intake. Multivariable regression analysis was performed to study the possible influence of confounding variables (i.e., BW, gender, cord blood pH, nutritional intake, morbidity) on linear measurements of structures that were significantly correlated with protein intake at the first week of life. The level of significance for all statistical tests was two-sided (*p* < 0.05).

### Ethics

The study was conducted in conformity with the World Medical Association Declaration of Helsinki for medical research involving human subjects. This article reports a part of the results of the study protocol that was approved by the ethics committee of Policlinico Umberto I, University La Sapienza of Rome (with number 5089). We asked parents for consent, and we collected anonymized data in the database.

## Results

**Figure 1:**
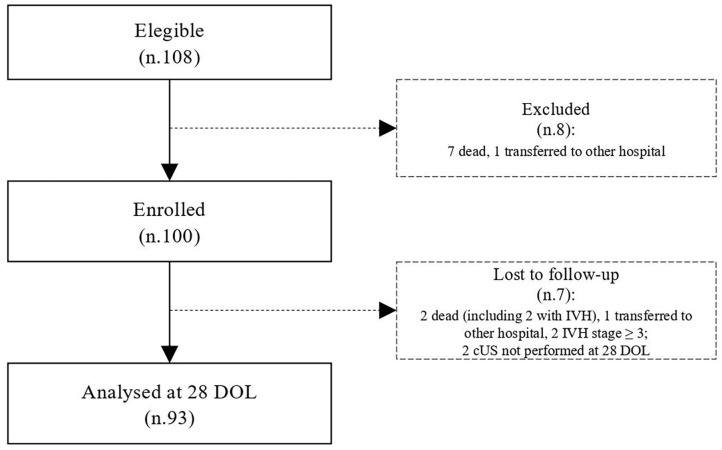
Flow chart. IVH, intraventricular hemorrhage; cUS, cranial Ultrasound; DOL, days of life.

**Figure 2:**
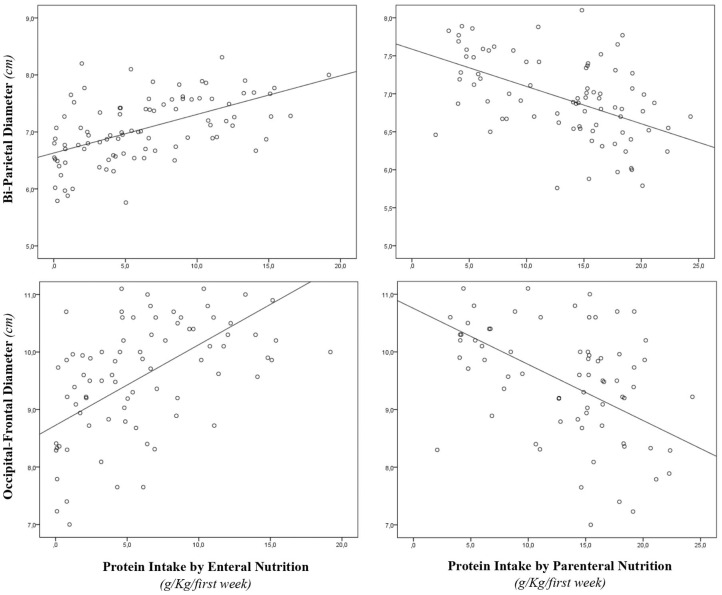
Correlation between brain measures at 28 days of life and early protein intake. Enternal nutrition: Bi-Parietal Diameter (*r* = 0.490, *p* < 0.001), Occipital-frontal diameter (*r* = 0.608, *p* < 0.001). Parameter nutrition: Bi-parietal diameter (*r* = −0.524, *p* < 0.001), Occipital-frontal diameter (*r* = −0.568, *p* < 0.001).

During the study period, we considered eligible 108 newborns. We enrolled 100 patients as shown in Figure 1. In Table 1, we reported the main demographic and clinical features of the study population. We observed a significant relation between head circumference and protein intake given by EN (*r* = 0.467, *p* < 0.001) and PN (*r* = −0.478, *p* < 0.001). We found a significant correlation between biparietal diameter, occipital–frontal diameter measured by cUS, and protein intake of the first week of life (Figure 2). Correlations between linear measurements of specific brain districts and early protein intake are reported in Table 2. In particular, length and genu of corpus callosum, caudate head (right and left), transverse diameter of cerebellum, and cerebellar vermis (height and width) were positively correlated with EN protein intake (Table 2). Body of corpus callosum, caudate head bilaterally, and cerebellum measures were negatively related with PN protein intake (Table 2). When we analyzed the total amount (EN + PN) of protein intake received in the first week of life, we observed a negative correlation with caudate head bilaterally and with vermis width (Table 2).

**Table 1:** Clinical characteristics of study population.

***N* = 100**	
Gestational age, weeks	29 ± 2
Birth weight, g	1,274 ± 363
Male sex, *n* (%)	55 (55)
Cesarean section, *n* (%)	88 (88)
Twins, *n* (%)	30 (30)
Antenatal steroids,^a^ *n* (%)	78 (78)
1-min Apgar score	6 ± 2
5-min Apgar score	8 ± 1
pH at birth	7.3 ± 0.1
Temperature at the 1st hour, °C	36.2 ± 0.5
Mortality, *n* (%)	3 (3)
Invasive mechanical ventilation, *n* (%)	22 (22)
NEC, *n* (%)	4 (4)
BPD, *n* (%)	4 (4)
IVH, *n* (%)	5 (5)
PLV, *n* (%)	1 (1)
ROP, *n* (%)	6 (6)
Sepsis proven by positive cultures, *N* (%)	10 (10)
Anemia of prematurity, *n* (%)	22 (22)
Full enteral feeding, days of life	15 ± 13
Star of enteral nutrition, days of life	1 ± 1
Duration of parenteral nutrition, days	15 ± 14

Data were expressed as mean ± standard deviation, when not specified.

a *Intramuscular steroids cycle in two doses of 12 mg over a 24-h period. NEC, necrotizing enterocolitis Bell stage ≥2); BPD, bronchopulmonary dysplasia; IVH intraventricular hemorrhage; PLV, periventricular leukomalacia; ROP, retinopathy of prematurity)*.

**Table 2:** Correlations between cerebral structures linear measures and protein intake.

	**Protein intake in the 1st week of life**		
**Measures^**a**^**	**By EN** **(g/kg**	**By PN** **(g/kg**	**Total** **(g/kg**
	**first week)**	**first week)**	**first week)**
**Corpus callosum**			
Length	0.293^*^	−0.252	−0.059
Body	0.244	−0.276^*^	−0.198
Genu	0.301^*^	−0.252	0.047
Splenium	0.169	−0.177	−0.105
**Caudate head**			
Right	0.528^**^	−0.613^**^	−0.468^**^
Left	0.364^**^	−0.444^**^	−0.372^**^
**Cerebellum**			
Transverse diameter	0.440^**^	−0.403^**^	−0.148
Vermis height	0.356^**^	−0.274*	0.001
Vermis width	0.377^**^	−0.462^**^	−0.387^**^

a Measured at 28 days of life.

* p < 0.05;

** *p < 0.01*.

Multivariate regression analysis showed that many linear measurements of brain structures at 28 DOL (occipital–frontal diameter, caudate heads, and cerebellar vermis) depended on nutritional intake (Table 3). Of note, we observed a different relation with EN (positive) and PN (negative).

**Table 3:** Multivariate analysis of covariate influencing cerebral measures at 28 days of life in preterm newborns.

**Dependent variables^**a**^**	**Covariates (model 1)**					**Covariates (model 2)**				
	**Birth**	**Male**	**pH at**	**Morbidity^**§**^**	**EN protein**	**Birth**	**Male**	**pH at**	**Morbidity^**§**^**	**PN protein**
	**weight**	**sex**	**birth**		**intake 1^**st**^ w**	**weight**	**sex**	**birth**		**intake 1st w**
Biparietal diameter	0.515^*^	−0.025	0.112	0.094	0.191	0.557^*^	0.039	0.130	0.091	−0.145
Occipital–frontal diameter	0.204	0.016	−0.023	−0.109	0.402^*^	0.319^*^	−0.051	−0.004	−0.139	−0.278^*^
Corpus callosum, length	0.106	0.080	−0.125	−0.213^*^	0.153	0.169	0.046	−0.141	−0.255^*^	−0.082
Corpus callosum, body	0.130	−0.082	0.235^*^	−0.106	0.036	0.062	−0.111	0.235^*^	−0.104	−0.121
Caudate head width, right	0.247^*^	0.076	0.079	0.080	0.305^*^	0.133	0.073	0.054	0.117	−0.488^*^
Caudate head width, left	0.238	−0.031	0.126	0.230^*^	0.210	0.072	−0.004	0.090	0.227^*^	−0.427^*^
Cerebellum transverse diameter	0.320^*^	0.233^*^	0.017	−0.004	0.164	0.468^*^	0.165	0.037	−0.003	−0.110
Cerebellar vermis, height	−0.073	0.209	−0.107	0.010	0.300^*^	0.070	0.117	−0.148	−0.078	−0.247
Cerebellar vermis, width	0.038	0.081	0.019	−0.083	0.271^*^	0.007	0.052	0.002	−0.171	−0.342^*^

a Measured at 28 days of life.

§ Necrotizing enterocolitis and/or sepsis proven by positive cultures and/or bronchopulmonary dysplasia.

* *p < 0.05. 1st w, first week of life; EN, enteral nutrition; PN, parenteral nutrition*.

Biparietal diameter, corpus callosum, and cerebellum transverse diameter depended on covariates (i.e., BW, sex, pH at birth, morbidity), but not by protein intake (Table 3).

Forty-five newborns were nourished mainly by PN in the first week of life. In this subpopulation, we found that left caudate head and cerebellar vermis width were smaller in subjects receiving high protein intake compared with those receiving low protein intake at 28 DOL, but not at T0 (Table 4, Table S2). In Table S3, we reported the occurrence of PN complications according to protein regimen.

**Table 4:** Effects of different protein intake in parenteral nutrition on cerebral measures.

**General**	**High protein**	**Low protein**	***p***
**characteristics**	**regimen**	**regimen**	
Number	23	22	
Gestational age at birth, weeks	27.6 ± 2.1	28.6 ± 2.5	0.161
Birth weight, g	1,014 ± 287	1,118 ± 320	0.763
Male sex, *n* (%)	13 (56.5)	14 (63.6)	1.000
Cesarean section, *n* (%)	21 (91.3)	18 (81.8)	0.311
Twins, *n* (%)	7 (30.4)	6 (27.3)	1.000
Antenatal steroids,^a^ *n* (%)	15 (65.2)	19 (86.4)	0.165
1-min Apgar score	5 ± 2	5 ± 2	0.369
5-min Apgar score	8 ± 1	8 ± 1	0.859
pH at birth	7.2 ± 0.1	7.3 ± 0.1	0.387
Temperature at the 1st hour, °C	36.0 ± 0.4	36.2 ± 0.5	0.137
Invasive mechanical ventilation, *n* (%)	8 (34.8)	10 (45.5)	0.550
NEC, *n* (%)	0 (0)	1 (4.5)	0.489
Sepsis, *n* (%)	5 (21.7)	2 (9.1)	0.414
BPD, *n* (%)	3 (13.0)	0 (0)	0.233
IVH, *n* (%)	1 (4.3)	4 (18.2)	0.187
IVH stage ≥3, *n* (%)	0 (0)	3 (13.6)	0.109
PLV, *n* (%)	0 (0)	0 (0)	-
ROP, *n* (%)	3 (13.0)	13 (13.6)	0.646
Anemia of prematurity, *n* (%)	10 (43.5)	8 (36.4)	0.428
Mortality, *n* (%)	1 (4.3)	1 (4.5)	1.000
Full enteral feeding, days of life	22 ± 16	21 ± 11	0.893
Star of enteral nutrition, days of life	2 ± 2	2 ± 2	0.343
Duration of parenteral nutrition, days	21 ± 16	20 ± 11	0.807
**Cerebral measures**^**b**^			
Biparietal diameter	66 ± 6	68 ± 6	0.226
Occipital–frontal diameter	90 ± 14	89 ± 10	0.697
Head circumference	282 ± 29	295 ± 21	0.188
Corpus callosum			
Length	38 ± 5	39 ± 3	0.578
Body	2 ± 0	2 ± 0	0.186
Genu	3 ± 1	3 ± 1	0.883
Splenium	3 ± 1	3 ± 1	0.167
Caudate head			
Right	5 ± 1	5 ± 1	0.101
Left	5 ± 1	6 ± 1	0.037
Cerebellum			
Transverse diameter	42 ± 5	43 ± 5	0.624
Vermis height	10 ± 2	10 ± 2	0.975
Vermis width	7 ± 1	8 ± 2	0.049

Data were expressed as mean ± standard deviation, when not specified. After percentile calculation, we considered 19.4 g/kg per week (50°pc) the cutoff value to classify protein regimen intake as “high” or “low”; an intramuscular steroids cycle in two doses of 12 mg over a 24-h period.

a Measured in mm at 28 days of life.

b *CRIB II: Clinical Risk Index for Babies, without temperature measures. NEC, necrotizing enterocolitis Bell stage ≥2; BPD, bronchopulmonary dysplasia; IVH, intraventricular hemorrhage; PLV, periventricular leukomalacia; ROP, retinopathy of prematurity*.

## Discussion

Brain development in neonatal life depends on early protein intake. We demonstrate that the route of protein administration may have different impact on cerebral measurements. Enteral intake has a positive effect, whereas high dose of amino acids by PN seems to affect the size of such brain structures during neonatal life.

Available evidences reported inconclusive results on the influence of early protein intake on the brain measurements (25). Differences between protein intake administered through either EN or PN have not been described in a recent meta-analysis including 21 randomized controlled trial (RCT) (25). In an RCT, comparing two groups of adolescents with a history of prematurity who were assigned to a standard- or high-EN protocol in the early postnatal life, those fed with preterm formula (protein 2 g/dL, 80 kcal/dL) showed significantly larger caudate volumes compared with babies fed by standard term formula (protein 1.4 g/dL, 68 kcal/dL) (26). The study is focused on the sole effect of enteral protein intake, whereas the role of PN was not investigated. Also, among cohort studies in this field, the relation between protein intake in PN and early cerebral growth was not clarified (5, 27, 28). Inconclusive results were also reported by studies that investigated the effects of early nutritional intake on brain maturation, instead of cerebral growth. Strømmen et al. (29) in an RCT reported a negative relation between aggressive nutrition and regional white matter mean diffusivity to TEA. No definite conclusions were drawn by Beauport et al. (30), which investigated the effects of total amounts of nutrients received in early life, without comparing the impact of protein intake given by PN separately by those of EN. To the best of our knowledge, we evaluate, for the first time, separately the role of route of administration of recommended protein intake on the brain in neonatal age. Our results underline the importance of protein intake on cerebral growth but, at the same time, suggest caution in the administration of high doses of amino acid in the first week of life through PN.

We hypothesize a number of mechanisms that may support the negative relation between PN intake and brain size. It is worth mentioning that newborns fed with higher PN are exposed to an environment, which limits infant's opportunity to develop their regulatory capacities (26). Furthermore, the side effects associated with aggressive PN (i.e., hyperglycemia, hypoglycemia, metabolic acidosis, elevated serum blood urea nitrogen, high plasma ammonia concentrations, hyperkalemia) may affect brain development (31). Recent studies have reported data on high protein intake delivered by PN leading to increased levels of specific amino acids, potentially toxic (e.g., glycine, phenylalanine, methionine) (32). It has also been described that insufficiency of amino acids, classified as essential, could represent a major limit for cerebral growth (33). Finally, an imbalance of amino acid levels may adversely affect neurotransmitters metabolism (32). In parallel, the positive effect of EN could be explained by the better composition of protein delivered through EN that may avoid the side effects PN related to the potential amino acids toxicity and by the balance of essential and non-essential amino acids.

We observed significant effects of protein intake mainly on caudate and cerebellar vermis, which are known to be a very vulnerable part of the brain in preterm infants (26). Preterm babies showing later neurological problems frequently present damage of these districts. We believe that protein intake may affect the development of this district in a crucial phase of cerebral growth. Dopamine is a main neurotransmitter of neonatal brain (34). The caudate structure is highly innervated by dopamine neurons (35). The direct precursor of dopamine, l-DOPA, is synthesized from the nonessential amino acid tyrosine (36). Recently, Mayes et al. (37) reported that hyperalimentation by PN can result in a paradoxical fall of tyrosine levels. Low tyrosine levels, affecting dopamine synthesis, may impair the development of brain districts that directly depend by dopamine neurons. However, further studies are advocated to clarify the relation between protein intake and dopamine metabolism in cerebral growth.

Despite being interesting, the results of this study should be interpreted taking into account specific limitations. This is a single-center observational study. We know that the cUS is a highly operator-dependent imaging modality. In order to limit associated bias, two different physicians performed a series of scans using cUS, and each measurement was recorded only after an agreement between the two investigators. In addition, the physicians who performed the cUS were unaware of the nutritional intake received at the time of the cUS examination. To measure cerebral structures, we used cUS instead of MRI, considered the gold-standard technique for the study of brain volumes. To improve the accuracy, we collected and analyzed only measurements of the cerebral structures that were previously assessed in a comparative study between MRI and cUS (6). The use of cUS allowed us to perform serial measurements of brain structures, avoiding issues of transporting to the radiological service and the sedation during the scan session for critical babies. Finally, no data on long-term neurodevelopment were analyzed in this study. Thus, it is not possible to establish if the results observed at 28 DOL on brain measurements may have consequences on the long-term neurodevelopment. Finally, comparison between high and low protein intake regimen was performed on a subpopulation of newborns. This may affect the generalizability of the results.

In conclusion, we observed that the administration of high doses of protein by PN is harmful for premature babies, in line with recent studies on pediatric population, which suggest avoiding aggressive nutritional practice in critically ill subjects (38). Positive impact on brain development encourages the administration of recommended protein intake mainly by EN. When EN is not possible, a beneficial undernutrition by parenteral route could be considered as a safe option, particularly in the first DOL. A challenging issue that should be addressed by further research is to establish what are the nutritional strategies promoting growth and brain development without additional risk for preterm babies.

## Data Availability Statement

The raw data supporting the conclusions of this article will be made available by the authors, without undue reservation.

## Ethics Statement

The studies involving human participants were reviewed and approved by the Ethics Committee of Policlinico Umberto I, University La Sapienza of Rome (with number 5089). Written informed consent to participate in this study was provided by the participants' legal guardian/next of kin.

## Author Contributions

GT, MCDN, GB, RC, and MDeC were responsible for the study design. GT, MCDN, GB, MDiC, and SC were responsible for the literature search and manuscript drafting. GT, MCDN, GB, MDiC, RC, SC, PP, MU, CG, BR, AR, GS, and MDeC were responsible for critical revision of the manuscript. All authors contributed to the article and approved the submitted version.

## Conflict of Interest

The authors declare that the research was conducted in the absence of any commercial or financial relationships that could be construed as a potential conflict of interest.

## References

[1] Kumar RK Singhal A Vaidya U Banerjee S Anwar F Rao S. Optimizing nutrition in preterm low birth weight infants—consensus summary. Front Nutr. (2017) 4:20. 10.3389/fnut.2017.00020 28603716 PMC5445116

[2] Hay WW. Aggressive nutrition of the preterm infant. Curr Pediatr Rep. (2013) 1:229–39. 10.1007/s40124-013-0026-4 24386613 PMC3875345

[3] van Goudoever JB Carnielli V Darmaun D Sainz de Pipaon M Braegger C Bronsky J. ESPGHAN/ESPEN/ESPR/CSPEN guidelines on pediatric parenteral nutrition: amino acids. Clin Nutrit. (2018) 37:2315–23. 10.1016/j.clnu.2018.06.945 30100107

[4] Senterre T Terrin G De Curtis M Parenteral nutrition in premature infants In: *Pediatric Gastroenterology, Hepatology and Nutrition: A Comprehensive Guide To Practice*. Cham: Springer (2016). p. 73–86. 10.1007/978-3-319-17169-2_7

[5] Coviello C Keunen K Kersbergen KJ Groenendaal F Leemans A Peels B. Effects of early nutrition and growth on brain volumes, white matter microstructure, and neurodevelopmental outcome in preterm newborns. Pediatr Res. (2018) 83:102–110. 10.1038/pr.2017.227 28915232

[6] Leijser LM Srinivasan L Rutherford MA Counsell SJ Allsop JM Cowan FM. Structural linear measurements in the newborn brain: accuracy of cranial ultrasound compared to MRI. Pediatr Radiol. (2007) 37:640–8. 10.1007/s00247-007-0485-2 17486330

[7] Dudink J Jeanne Steggerda S Horsch S eurUS. brain group. State-of-the-art neonatal cerebral ultrasound: technique and reporting. Pediatr Res. (2020) 87:3–12. 10.1038/s41390-020-0776-y PMC7098885 32218539

[8] Beijst C Dudink J Wientjes R Benavente-Fernandez I Groenendaal F Brouwer MJ. Two-dimensional ultrasound measurements vs. magnetic resonance imaging-derived ventricular volume of preterm infants with germinal matrix intraventricular haemorrhage. Pediatr Radiol. (2020) 50:234–41. 10.1007/s00247-019-04542-x 31691845 PMC6978291

[9] Horsch S Bengtsson J Nordell A Lagercrantz H Ådén U Blennow M. Lateral ventricular size in extremely premature infants: 3D MRI confirms 2D ultrasound measurements. Ultrasound Med Biol. (2009) 35:360–6. 10.1016/j.ultrasmedbio.2008.09.006 19056162

[10] Benavente-Fernandez I Lubián-Gutierrez M Jimenez-Gomez G Lechuga-Sancho AM Lubián-López SP Neonatal Neurology Foundation (Fundación Nene). Ultrasound lineal measurements predict ventricular volume in posthaemorrhagic ventricular dilatation in preterm infants. Acta Paediatr. (2017) 106:211–7. 10.1111/apa.13645 27783429

[11] Hintz SR Barnes PD Bulas D Slovis TL Finer NN Wrage LA. Neuroimaging and neurodevelopmental outcome in extremely preterm infants. Pediatrics. (2015) 135:e32–e42. 10.1542/peds.2014-0898 25554820 PMC4279063

[12] Canani RB Terrin G. Recent progress in congenital diarrheal disorders. Curr Gastroenterol Rep. (2011) 13:257–64. 10.1007/s11894-011-0188-6 21494839

[13] Salvia G Cascioli CF Ciccimarra F Terrin G Cucchiara S A case of protein-losing enteropathy caused by intestinal lymphangiectasia in a preterm infant. Pediatrics. (2001) 107:416–7. 10.1542/peds.107.2.416 11158480

[14] Passariello A. Diarrhea in neonatal intensive care unit. WJG. (2010) 16:2664. 10.3748/wjg.v16.i21.2664 20518089 PMC2880780

[15] Whitelaw A. Intraventricular haemorrhage and posthaemorrhagic hydrocephalus: pathogenesis, prevention and future interventions. Semin Neonatol. (2001) 6:135–46. 10.1053/siny.2001.0047 11483019

[16] Parry G Tucker J Tarnow-Mordi W. CRIB II: an update of the clinical risk index for babies score. Lancet. (2003) 361:1789–1791. 10.1016/S0140-6736(03)13397-1 12781540

[17] Terrin G Boscarino G Di Chiara M Iacobelli S Faccioli F Greco C. Nutritional intake influences zinc levels in preterm newborns: an observational study. Nutrients. (2020) 12:529. 10.3390/nu12020529 32093077 PMC7071515

[18] Terrin G Coscia A Boscarino G Faccioli F Di Chiara M Greco C. Long-term effects on growth of an energy-enhanced parenteral nutrition in preterm newborn: a quasi-experimental study. PLoS ONE. (2020) 15:e0235540. 10.1371/journal.pone.0235540 32628715 PMC7337335

[19] Terrin G Passariello A Canani RB Manguso F Paludetto R Cascioli C. Minimal enteral feeding reduces the risk of sepsis in feed-intolerant very low birth weight newborns. Acta Paediatrica. (2009) 98:31–5. 10.1111/j.1651-2227.2008.00987.x 18727685

[20] Berni Canani R Passariello A Buccigrossi V Terrin G Guarino A. The nutritional modulation of the evolving intestine. J Clin Gastroenterol. (2008) 42:S197–200. 10.1097/MCG.0b013e31817da155 18685515

[21] Graça AM Geraldo AF Cardoso K Cowan FM. Preterm cerebellum at term age: ultrasound measurements are not different from infants born at term. Pediatr Res. (2013) 74:698–704. 10.1038/pr.2013.154 24002327

[22] Davies MW Swaminathan M Betheras FR. Measurement of the transverse cerebellar diameter in preterm neonates and its use in assessment of gestational age. Australas Radiol. (2001) 45:309–12. 10.1046/j.1440-1673.2001.00926.x 11531754

[23] Imamoglu EY Gursoy T Ovali F Hayran M Karatekin G. Nomograms of cerebellar vermis height and transverse cerebellar diameter in appropriate-for-gestational-age neonates. Early Hum Dev. (2013) 89:919–23. 10.1016/j.earlhumdev.2013.10.001 24183100

[24] da Graça ALFM Cardoso KRV da Costa JMFP Cowan FM. Assessment of gestational age using cerebellar measurements at cranial ultrasound: what is the best approach? Early Hum Dev. (2013) 89:1–5. 10.1016/j.earlhumdev.2012.07.008 22835598

[25] Osborn DA Schindler T Jones LJ Sinn JK Bolisetty S. Higher versus lower amino acid intake in parenteral nutrition for newborn infants. Cochr Database System Rev. (2018) 3:1465–858. 10.1002/14651858.CD005949.pub2 29505664 PMC6494253

[26] Isaacs EB Gadian DG Sabatini S Chong WK Quinn BT Fischl BR. The effect of early human diet on caudate volumes and IQ. Pediatr Res. (2008) 63:308–14. 10.1203/PDR.0b013e318163a271 18287970

[27] Power VA Spittle AJ Lee KJ Anderson PJ Thompson DK Doyle LW. Nutrition, growth, brain volume, and neurodevelopment in very preterm children. J Pediatrics. (2019) 215:50–5.e3. 10.1016/j.jpeds.2019.08.031 31561956

[28] Schneider J Fischer Fumeaux CJ Duerden EG Guo T Foong J Graz MB. Nutrient intake in the first two weeks of life and brain growth in preterm neonates. Pediatrics. (2018) 141:e20172169. 10.1542/peds.2017-2169 29440285

[29] Strømmen K Blakstad EW Moltu SJ Almaas AN Westerberg AC Amlien IK. Enhanced nutrient supply to very low birth weight infants is associated with improved white matter maturation and head growth. Neonatology. (2015) 107:68–75. 10.1159/000368181 25401387

[30] Beauport L Schneider J Faouzi M Hagmann P Hüppi PS Tolsa J-F. Impact of early nutritional intake on preterm brain: a magnetic resonance imaging study. J Pediatrics. (2017) 181:29–36.e1. 10.1016/j.jpeds.2016.09.073 27837953

[31] Stensvold HJ Strommen K Lang AM Abrahamsen TG Steen EK Pripp AH. Early enhanced parenteral nutrition, hyperglycemia, and death among extremely low-birth-weight infants. JAMA Pediatr. (2015) 169:1003. 10.1001/jamapediatrics.2015.1667 26348113

[32] te Braake FWJ van den Akker CHP Riedijk MA van Goudoever JB. Parenteral amino acid and energy administration to premature infants in early life. Sem Fetal Neonatal Med. (2007) 12:11–8. 10.1016/j.siny.2006.10.002 17142119

[33] Makrides M Gibson RA McPhee AJ Collins CT Davis PG Doyle LW. Neurodevelopmental outcomes of preterm infants fed high-dose docosahexaenoic acid: a randomized controlled trial. JAMA. (2009) 301:175. 10.1001/jama.2008.945 19141765

[34] Volpe JJ Volpe JJ eds. Volpe's Neurology of the Newborn. Sixth edition Philadelphia, PA: Elsevier (2018).

[35] White NM. Some highlights of research on the effects of caudate nucleus lesions over the past 200 years. Behav Brain Res. (2009) 199:3–23. 10.1016/j.bbr.2008.12.003 19111791

[36] Musacchio JM Chapter 1: enzymes involved in the biosynthesis degradation of catecholamines. In: *Inverson L. Biochemistry of Biogenic Amines*. Boston, MA: Springer (2013). p. 1–35. 10.1007/978-1-4684-3171-1_1

[37] Mayes K Tan M Morgan C. Effect of hyperalimentation and insulin-treated hyperglycemia on tyrosine levels in very preterm infants receiving parenteral nutrition. JPEN J Parenter Enteral Nutr. (2014) 38:92–8. 10.1177/0148607112467036 23169901

[38] Vanhorebeek I Verbruggen S Casaer MP Gunst J Wouters PJ Hanot J. Effect of early supplemental parenteral nutrition in the paediatric ICU: a preplanned observational study of post-randomisation treatments in the PEPaNIC trial. Lancet Respir Med. (2017) 5:475–83. 10.1016/S2213-2600(17)30186-8 28522351

