# Supplementary material for: Early Protein Intake Influences Neonatal Brain Measurements in Preterms: An Observational Study
Source: Front Neurol. 2020 Aug 26;11:885. doi: 10.3389/fneur.2020.00885 (PMC7479306; doi:10.3389/fneur.2020.00885)
Supplement: Supplementary file 1 [file Table_1.DOCX]

Supplementary Material

# Supplementary Tables

**Table S1**. Parenteral Nutrition protocol.

|  | Birth weight < 1000 g | Birth weight ≥ 1000 g |
| --- | --- | --- |
| Energy *(kcal/kg/day)* |  |  |
| Starting dose | 45 | 45 |
| *At day of life* | 0 | 0 |
| Target dose | 105 | 100 |
| *At day of life* | 7 | 7 |
| Proteins *(g/kg/day)* |  |  |
| Starting dose | 2.0 | 2.0 |
| *At day of life* | 0 | 0 |
| Target dose | 4.0 | 3.5 |
| *At day of life* | 7 | 7 |
| Dextrose *(g/kg/day)* |  |  |
| Starting dose | 7.0 | 7.0 |
| *At day of life* | 0 | 0 |
| Target dose | 14.0 | 14.5 |
| *At day of life* | 7 | 7 |
| Lipids *(g/kg/day)* |  |  |
| Starting dose | 1.0 | 1.0 |
| *At day of life* | 0 | 0 |
| Target dose | 3.5 | 3.0 |
| *At day of life* | 7 | 7 |

**Table S2.** Basal characteristics of newborns receiving different protein intake by parenteral nutrition.

| *Cerebral Measures (mm)* | **High**  **Protein Regimen** | **Low**  **Protein Regimen** | ***p*** |
| --- | --- | --- | --- |
| **Bi-Parietal Diameter** | 61.3 ± 7.9 | 64.7 ± 6.5 | 0.134 |
| **Occipital-Frontal Diameter** | 75.9 ± 10.9 | 74.8 ± 7.4 | 0.697 |
| **Head Circumference** | 256.9 ± 24.0 | 261.3 ± 21.9 | 0.536 |
| **Corpus Callosum** |  |  |  |
| *Length* | 34.7 ± 3.9 | 35.8 ± 4.7 | 0.390 |
| *Body* | 2.0 ± 0.4 | 2.1 ± 0.5 | 0.691 |
| *Genu* | 3.1 ± 0.5 | 3.2 ± 0.7 | 0.498 |
| *Splenium* | 3.3 ± 0.8 | 2.9 ± 0.8 | 0.106 |
| **Caudate Head** |  |  |  |
| *Right* | 4.3 ± 1.1 | 4.9 ± 1.1 | 0.101 |
| *Left* | 4.3 ± 0.9 | 4.9 ± 1.0 | 0.062 |
| **Cerebellum** |  |  |  |
| *Transverse diameter* | 33.8 ± 5.3 | 36.0 ± 8.8 | 0.315 |
| *Vermis Height* | 7.8 ± 1.5 | 8.1 ± 1.8 | 0.576 |
| *Vermis Width* | 5.5 ± 1.2 | 6.3 ± 1.5 | 0.078 |

**Notes.** After percentile calculation we considered 19.4 g/Kg/week the cut off value to classify protein regimen intake as “high” or “low”; Data were expressed as mean ± standard deviation, when not specified.

**Table S3.** Side effects associated with the use of parenteral nutrition in enrolled newborns, classified by different protein intake given by parenteral nutrition.

| *Side effects* | **High**  **Protein Regimen** | **Low**  **Protein Regimen** | ***p*** |  |
| --- | --- | --- | --- | --- |
| **Hyperglycaemia** | 12 (52.2) | 9 (40.9) | 0.554 | |
| **Hypoglycaemia** | 3 (13.0) | 2 (9.1) | 0.522 | |
| **Hypercalemia** | 11 (47.8) | 14 (63.6) | 0.373 | |
| **Hypocalcemia** | 4 (17.4) | 8 (36.4) | 0.189 | |
| **Hyperphosphatemia** | 1 (4.3) | 3 (13.6) | 0.287 | |
| **Hypophosphatemia** | 6 (26.1) | 5 (22.7) | 1.000 | |
| **Hyperuremia** | 11 (47.8) | 15 (68.2) | 0.231 | |
| **Hypouremia** | 7 (30.4) | 3 (13.6) | 0.160 | |
| **Metabolic Acidosis** | 8 (34.8) | 10 (45.5) | 0.550 | |

**Notes.** After percentile calculation we considered 19.4 g/Kg/week the cut off value to classify protein regimen intake as “high” or “low”; Data were expressed as No. (%).

Glycaemia was defined hyper > 180 mg/dl and hypo < 38 md/dl. Calcemia was defined hyper >2.4 mmol/l or > 11 mg/dl and hypo < 1.6 mmol/l or < 7.5 mg/dl. Phosphatemia was defined hyper > 3.1 mmol/l or > 9.6 md/dl and hypo < 1.6 mmol/l or < 5 mg/dl. Uremia was defined hyper > 5 mmol/l or > 14 mg/dl and hypo < 2.9 mmol/l or < 8.1 mg/dl. Metabolic acidosis was defined BE< 10 mmol or pH < 7.25 with pCO2 > 50.
