# Supplementary material for: Early Protein Intake Influences Neonatal Brain Measurements in Preterms: An Observational Study
Source: Front Neurol. 2020 Aug 26;11:885. doi: 10.3389/fneur.2020.00885 (PMC7479306; doi:10.3389/fneur.2020.00885)
Supplement: Supplementary file 2 [file Data_Sheet_1.PDF]

SISTEMA SANITARIO REGIONALE
AZIENDA OSPEDALIERA UNIVERSITARIA
POLICLINICO UMBERTO I

SAPIENZA
UNIVERSITÀ DI ROMA

PRESA VISIONE DEL PARERE FAVOREVOLE DEL COMITATO ETICO DELL'UNIVERSITA' "SAPIENZA"

SI autorizza la conduzione presso questa Struttura dello studio:

"Effetti della nutrizione sulla crescita e sullo sviluppo neuropsicomotorio del neonato pretermine."

approvato dal Comitato Etico dell' Università "Sapienza" nella seduta del 13.09.18 – Rif. 5089 da attuarsi sotto la responsabilità dello Sperimentatore Principale: *Prof. Gianluca TERRIN*

Il Direttore Sanitario
Prof. Ferdinando ROMANO

Il Direttore Amministrativo
Dott. Massimiliano GERLI

Il Direttore Generale
Dott. Vincenzo PANELLA

19 SET. 2018

Roma, 14.09.18
