# Supplementary material for: Early Protein Intake Influences Neonatal Brain Measurements in Preterms: An Observational Study
Source: Front Neurol. 2020 Aug 26;11:885. doi: 10.3389/fneur.2020.00885 (PMC7479306; doi:10.3389/fneur.2020.00885)
Supplement: Supplementary file 3 [file Data_Sheet_2.PDF]

SISTEMA SANITARIO REGIONALE

AZIENDA OSPEDALIERA UNIVERSITARIA
POLICLINICO UMBERTO I

SAPIENZA
UNIVERSITÀ DI ROMA

ACKNOWLEDGMENT OF THE ETHICAL COMMITTEE APPROVAL "SAPIENZA" UNIVERSITY

We **ATHORIZE** in this hospital structure the study:

"Impact of nutrition on body growth and neurodevelopment in preterm newborn."

approved by the Ethics Committee of the "Sapienza" University in the session of 13.09.18 – Ref. 5089 under the responsibility of Prof. Gianluca TERRIN as Principal Researcher.

The Medical Director
Prof. Ferdinando ROMANO

Head of administration
Dott. Massimiliano GERLI

The Hospital Administrative Director
Dott. Vincenzo PANELLA

Rome, 14.09.18

Azienda Policlinico Umberto I
Viale del Policlinico, 155 ✉ 00161 Roma
Centralino (+39) 06 49971
C.F. e P.IVA 05865511009
www.policlinicoumberto1.it
